# Reversal of the Symptoms of Diabetic Neuropathy through Correction of Vitamin D Deficiency in a Type 1 Diabetic Patient

**DOI:** 10.1155/2012/165056

**Published:** 2012-12-12

**Authors:** David S. H. Bell

**Affiliations:** Southside Endocrinology, University of Alabama Medical School, Suite 130, 3928 Montclair Road, Birmingham, AL 35213, USA

## Abstract

Vitamin D deficiency has been associated with both type 1 and type 2 diabetes as well as both the microvascular and macrovascular complications of diabetes. Vitamin D deficiency has been shown to be more common in diabetic patients who have symptoms of distal symmetrical polyneuropathy. In addition, vitamin D deficiency has been associated with a lower pain threshold which increases when vitamin D deficiency is corrected. Herein, I describe a type 1 diabetic patient with neuropathic symptoms so severe that he could not work and for which he needed narcotics for pain management and whose symptoms improved dramatically with correction of the vitamin D deficiency. To my knowledge, this is the first report of an improvement in severe symptoms of diabetic neuropathy with correction of vitamin D deficiency in a single patient.

## 1. Introduction 

Vitamin D deficiency has in addition to bone disease been implicated as a factor or cofactor in the etiology of type 2 diabetes, type 1 diabetes and other autoimmune diseases, heart disease, and cancers [1]. In addition, vitamin D deficiency is often diagnosed in those with established diabetes and vitamin D replacement may prevent or delay the onset of diabetic complications [2]. In addition pain thresholds of multiple etiologies have been reported to be lowered with vitamin D deficiency and elevated when the vitamin D deficiency is corrected [3]. 

When distal symmetrical neuropathy is diagnosed in a diabetic patient, causes other than diabetes itself are seldom found. However, it is recommended that a paraproteinemia and vitamin B_12_ deficiency are eliminated with a plasma protein electrophoresis and vitamin B_12_ level. Vitamin B_12_ deficiency is common in patients who utilize metformin for treatment of their type 2 diabetes, and there is also a higher prevalence of pernicious anemia with type 1 diabetes. 

## 2. Clinical Presentation

 A 38-year-old type 1 (C-peptide < 0.01 ng/mL) diabetic patient had had diabetes for 27 years and neuropathic symptoms (tingling, burning, shooting pains, and paresthesias) in both hands and feet for 10 years with an escalation of the severity of symptoms occurring during the previous four years. 

 Initially, he had partial relief of his symptoms with tricyclics, gabapentin, and pregabalin. However, his symptoms became so severe that he had to stop working, and for pain control he required narcotic analgesia (oxycodone) which only marginally ameliorated his symptoms. Objective findings were typical of a small fiber neuropathy with preservation of reflexes and vibration sense with hyperesthesia to painful stimuli to the level of the ankles bilaterally. 

 Both vitamin B_12_ deficiency and a paraproteinemia were ruled out as a cause of his neuropathic symptoms, and his HbA1c was 7.0%. A 25-OH-vitamin D level was preformed because the patient was obese (6′2′′ 245 lbs) (BMI 31.5), and obesity as well as type 1 diabetes is often associated with low vitamin D levels [1]. Based on his 25-OH-vitamin D level which was 16.5 ng/dL he was started on 50,000 units of vitamin D_2_ weekly, and within two weeks his symptoms began to decrease. Within four weeks his symptoms had improved to a level at which he was able to discontinue his oxycodone and at that time his symptoms were controlled with clonazepam 2 mg daily and sinequan 150 mg hs. A repeat 25-hydroxy-vitamin D level after one month of therapy was 48 ng/dL. 

## 3. Discussion 

 If a proven vitamin deficiency exists then correction of the deficiency may be beneficial and not cause harm. However, if a vitamin level is in the normal range additional vitamin supplementation is of no value and can be harmful [1]. In this case, correction of the vitamin D deficiency was undertaken only to prevent the development of the protean manifestations of vitamin D deficiency and not to treat the symptoms of diabetic neuropathy [1]. However, to my surprise repletion of vitamin D resulted in a very significant improvement in his neuropathic symptoms. This to my knowledge is the first report of the symptoms of severe and disabling diabetic neuropathy being improved by correction of the vitamin D deficiency in an individual patient. 

 Neuropathy in some shape or form occurs in 60–70% of diabetic patients with 50% of these patients experiencing varying degrees of neuropathic pain which invariably results in a decreased quality of life [4]. The question which remains unanswered in this case is whether the correction of vitamin D deficiency simply resulted in a nonspecific elevation of the pain threshold as has been described by Plotnkoff et al. or was due to a specific pathological improvement in the affected nerves or was it due to a combination of elevating the pain threshold and an amelioration of nerve damage [3]. 

 Animal studies have shown that vitamin D deficiency is associated with low levels of neurotrophins (especially nerve growth factor) and defective neuronal calcium homeostasis. In addition vitamin D through its receptor modulates neuronal differentiation as well as neuronal growth and function [5]. In rats, the production of nerve growth factor which is required for the development and survival of both sympathetic and sensory neurons decreases in the presence of vitamin D deficiency. In fact, in vitamin D deficient diabetic animals correction of vitamin D deficiency resulted in an improvement in nerve growth factor production [6]. Decrease in neurotrophins and defective calcium homeostasis leaves the nerve vulnerable to toxins including hyperglycemia. As a result, a deficiency of vitamin D impairs nociceptor function, worsens nerve damage, and lowers the pain threshold [6]. 

 In humans in the National Health and Nutrition Examination Survey, vitamin D deficiency (defined as a level less than 30 ng/mL) in adults diabetes was associated with, after statistical correction for the HbA1c level, with the symptoms (numbness, pain, loss of feeling, and tingling in the hands and/or feet) of diabetic neuropathy [7]. 

 In another study vitamin D levels were not only inversely proportional to a neuropathy symptoms score but also showed a statistically significant (OR 3.47 95% CI 1.04–11.56 *P* = 0.04) association with slower nerve conduction velocities after correction for duration of diabetes and levels of HbA1c, LDL, and urinary albumin [8]. 

 Prospectively, a nonrandomized study of 51 type 1 diabetic subjects with painful diabetic neuropathy showed a 50% decrease in pain scores with vitamin D repletion [9]. However, the neuropathic pain level in this group was much less than the severe pain level described by this individual patient. In addition, in humans topical vitamin D application to the areas affected by neuropathy has been reported to relieve neuropathic symptoms [10].

 Therefore, this case study where severe neuropathic symptoms were corrected by restoring vitamin D levels into normal range validates the previous studies of less severe neuropathic symptoms [9, 10]. From the patient's and the clinician's viewpoint whether the improvement in symptoms is due to improvement in nerve damage or simply due to a nonspecific vitamin D induced elevation of the pain threshold is of little consequence and only of academic interest. 

 I would therefore propose that in all diabetic patients presenting with symptomatic or nonsymptomatic neuropathy a 25-OH vitamin D level is obtained and that if the 25-OH-vitamin D level is less than 30 ng/mL, therapy with vitamin D_2_ or D_3_ is initiated to elevate the 25-OH vitamin D level to a level of above 30 ng/mL. The correction of vitamin D deficiency cannot be harmful and has the potential to alleviate neuropathic symptoms and lower the need for medications especially narcotics with their often severe side effects. In addition with correction of vitamin D deficiency, there is the potential of arresting and perhaps reversing the progression of neuronal destruction. 
